# Morphological and Calcium Signaling Alterations of Neuroglial Cells in Cerebellar Cortical Dysplasia Induced by Carmustine

**DOI:** 10.3390/cells10071581

**Published:** 2021-06-23

**Authors:** Cynthia Alejandra Rodríguez-Arzate, Marianne Lizeth Martínez-Mendoza, Israel Rocha-Mendoza, Yryx Luna-Palacios, Jacob Licea-Rodríguez, Ataúlfo Martínez-Torres

**Affiliations:** 1 Instituto de Neurobiología (INB), Universidad Nacional Autónoma de México (UNAM), Campus Juriquilla, Querétaro 76230, QT, Mexico; ale_rodriguez@comunidad.unam.mx (C.A.R.-A.); mlmminb@comunidad.unam.mx (M.L.M.-M.); 2 Centro de Investigación Científica y de Educación Superior de Ensenada (CICESE), Carretera Ensenada-Tijuana, No. 3918, Zona Playitas, Ensenada 22860, BC, Mexico; irocha@cicese.mx (I.R.-M.); yluna@cicese.edu.mx (Y.L.-P.); jlicea@cicese.edu.mx (J.L.-R.); 3 Cátedras CONACYT, Centro de Investigación Científica y de Educación Superior de Ensenada (CICESE), Ensenada 22860, BC, Mexico

**Keywords:** astrocytes, Bergmann glia, clarity, light-sheet fluorescence microscopy

## Abstract

Cortical dysplasias are alterations in the organization of the layers of the brain cortex due to problems in neuronal migration during development. The neuronal component has been widely studied in experimental models of cortical dysplasias. In contrast, little is known about how glia are affected. In the cerebellum, Bergmann glia (BG) are essential for neuronal migration during development, and in adult they mediate the control of fine movements through glutamatergic transmission. The aim of this study was to characterize the morphology and intracellular calcium dynamics of BG and astrocytes from mouse cerebellum and their modifications in a model of cortical dysplasia induced by carmustine (BCNU). Carmustine-treated mice were affected in their motor coordination and balance. Cerebellar dysplasias and heterotopias were more frequently found in lobule X. Morphology of BG cells and astrocytes was affected, as were their spontaneous [Ca^2+^]^i^ transients in slice preparation and in vitro.

## 1. Introduction

Cortical dysplasia is a type of brain developmental disorder that is frequently associated with clinical manifestations such as epileptic seizures [1]. It is characterized by abnormal organization of the layers of the brain and the presence of heterotopias—clusters of cells that are not necessarily dysfunctional but rather in abnormal positions. Genetic and acquired factors give rise to cortical dysplasia and affect cell proliferation, migration and maturation during early development [2,3]. Although multiple causes of cortical dysplasias have been proposed and several epileptogenic mechanisms have been revealed, the etiology is currently unclear. In addition, there is scant information on cortical dysplasias in the cerebellum.

The neuronal component has been widely studied in experimental models of cortical dysplasia. Several abnormalities have been reported in these models, including laminar disorganization, cytomegalic neurons, heterotopic clusters of Cajal–Retzius cells, and dysmorphic pyramidal neurons of layers II and III [4] with physiological alterations that showed neuronal hyperexcitability and decreased sensitivity to γ-aminobutyric acid (GABA) [5,6]. In the hippocampus, heterotopic neurons have an increased response to N-methyl-d-aspartate (NMDA), generating currents with a reduced decay-time constant [7] and an enhanced long-term potentiation associated with an increase in excitability [8].

In contrast to what is known of the neuronal component, information about how glial brain cells are affected in cortical dysplasia is scant. A prominent component observed in focal cortical dysplasia are the balloon cells, which express glial fibrillary acidic protein (GFAP), an astrocyte intermediate filament [9,10]. Astrocytes seem to functionally adapt in patients with intractable epilepsy associated with focal cortical dysplasia, displaying changes in the expression of molecular components critical for astrocyte and neuronal communication, which are linked to modifications in the activation of intracellular Ca^2+^ cascades and neuron-glia interactions that contribute to epileptogenesis [11]. Another study of astrocytic function linked to cortical dysplasia showed reduced gap junction coupling between astrocytes in freeze-lesion-induced dysplastic neocortex [12].

In a previous study we assessed how the cerebellum was affected by prenatal exposure to carmustine, an alkylating agent used to reproduce anatomical features seen in human patients with cortical dysplasia. The cerebellum is the main motor coordination center in the central nervous system but also modulates cognitive processes such as language, perception and spatial memory [13,14]. The cortex of the cerebellum is divided into three cell layers: the outer molecular layer, the middle layer of Purkinje cells and the inner granular layer. Carmustine-treated mice exhibited disorganization in these cell layers, which was causally linked to changes in motor performance [15]. Furthermore, Bergmann glial cells (BG), whose soma is normally located in the Purkinje cell layer, were displaced and the complexity of their processes severely diminished. The effect on other types of glial cells was not explored in that study.

Cerebellar glial cells play an important role in various processes such as proliferation, migration and differentiation throughout development and maturation. Since they are closely associated with neurons, they are involved in the alteration of synaptogenesis, playing a critical role in brain diseases [16]. Astrocytes represent the most abundant type of glial cell in the central nervous system and participate in multiple biological functions [17], whereas BG cells are considered a specialized type of astrocyte derived from radial glia and unique to the cerebellum. They are found from very early stages of development, when they form a structural scaffold for the migration of granule cells during postnatal development, as well as in the dendritic growth of Purkinje cells [13].

In our previous report we found that carmustine induces dramatic changes in the organization of the cell layers of the cerebellum and in BG cells. Beyond this, little is known about how the function of BG cells and astrocytes is affected. Here we show by using light sheet fluorescence microscopy how the folia of the cerebellum are disorganized in the carmustine model of cortical dysplasia and provide evidence that calcium signaling between glial cells is abnormal and may be a potential source of disrupted normal synaptic transmission.

## 2. Materials and Methods

### 2.1. Animal Handling

All experimental manipulations, protocols and procedures were carried out according to the ethical policies for animal care and handling (INEU/SA/CB089) approved by the Bioethics committee of the Institute of Neurobiology of the National Autonomous University of Mexico, in accordance with national (NOM-062-ZOO-1999) and international guidelines (National Institutes of Health, USA). For this study we used the transgenic mouse GFAP-eGFP [18] that expresses the green fluorescent protein under the control of the GFAP promoter that directs the expression in astrocytes and Bergmann cells. We also used the CD1 mouse strain to isolate cerebellar astrocytes.

### 2.2. Experimental Model of Cortical Dysplasia

The experimental induction of cortical dysplasia was carried out on transgenic GFAP-eGFP and CD1 pregnant female mice on embryonic day 13 (E13, which is the peak of gliogenesis; E1 was considered the day in which the vaginal plug was observed) by intraperitoneal administration of carmustine (Sigma Aldrich, Saint-Louis, MO, USA) at a dose of 20 mg/kg. The solution was prepared by dissolving carmustine in 5% glucose at 4 mg/mL. For control animals, pregnant female mice were intraperitoneally injected with vehicle (5% glucose in sterile water) on the same embryonic day, according to the method previously reported [5,6]. The injected gestational females were housed in the INB-UNAM vivarium with 12:12 h light:dark cycles and allowed food and water *ad libitum*. The pups were used for the following studies. The day of birth of the pups was considered as postnatal day (P0).

### 2.3. Behavioral Tests

The impact on motor coordination and balance was assessed with a Rotarod. Transgenic GFAP-eGFP P30 male mice (control and carmustine treated) were evaluated on a rotarod apparatus (Series 8, IITC Life Science, Los Angeles, CA, USA) in two test sessions on consecutive days [19]. Each test session per day consisted of 3 training trials (5 rpm per minute), followed by 3 acceleration trials (acceleration rate of 40 rpm/min). The rod started at 5 rpm but gradually increased to 40 rpm. Before the first test session, mice were habituated to the room for 30 min. The test session began when the mice were gently placed on the rod and finished when the mice made an error. The error was considered as the inability of the mouse to keep walking on the rod (latency fall) evaluated by one of the following events: (1) The mouse fell down or (2) the mouse remained clung to the rod. The distance, time and maximum acceleration of each mouse in every trial was recorded and averaged for each session.

### 2.4. Tissue Clearing

Transgenic GFAP-eGFP P5–P7 male mice were anesthetized intraperitoneally with pentobarbital (30 mg/kg, Cheminova, Washington, NC, USA) and perfused with 0.9% NaCl followed by hydrogel solution. The hydrogel solution was kept on ice and prepared by dissolving 40 mL of acrylamide (40%, Bio-Rad Laboratories, Hercules, CA, USA), 10 mL of bis-acrylamide (2%, Bio-Rad Laboratories), 1 g of initiator VA-044 (0.25%, Alpha Laboratories, Toronto, ON, Canada), 40 mL of 10X PBS, 100 mL of 16% PFA in 1 L of dH_2_O. After perfusion, brains were removed and kept in hydrogel solution for 3 days at 4 °C [20]. The tissue was placed in a 15 mL Falcon tube that was completely filled with fresh hydrogel solution at 37 °C in a water bath for 3 h to induce the polymerization of hydrogel. Once the gel had polymerized, the brain was extracted by removing the excess of hydrogel. Three washes with clarifying solution were carried out for 24 h at 37 °C, on a shaker at 100 RPM. The clarifying solution was prepared by dissolving 12.36 g of boric acid (Sigma Aldrich, Saint-Louis, MO, USA), 40 g of sodium dodecyl sulfate (Sigma Aldrich, Saint-Louis, MO, USA) and made up to 1 L with dH_2_O, pH 7.5. Then, the tissue was placed on a shaker at 100 RPM at 37 °C for passive clarification [20]. Once clarified, the sample was washed four times with PBST for 30 min at 37 °C on a shaker at 80 RPM. The tissue was kept in glycerol (80%) for 24 h before imaging.

### 2.5. Light-Sheet Fluorescent Microscopy

Three-dimensional imaging of the cerebellum was performed using light-sheet fluorescence microscopy (LSFM). In LSFM, a light-sheet excites a single fluorescent plane within the sample, while the excited fluorescent plane is imaged through a microscope objective and a camera placed orthogonal to the excitation light-sheet. LSMF can perform wide-field optical sectioned (tomographic-like) imaging with good optical resolution over volumetric samples at different length scales [21,22,23,24].

#### 2.5.1. Image Acquisition

Two different LSFM systems were implemented under the selective plane illumination microscopy (SPIM) configuration. The first system, named macro-SPIM, was utilized to image large fields of view (FoV) with moderate optical sectioning and resolution. The second system, named micro-SPIM, took images at smaller FoV but with optical sectioning and resolution comparable to confocal laser microscopy. Both systems utilized a continuous-wave laser (Obis, Coherent, Santa Clara, CA, USA), emitting at 488 nm wavelength for fluorescence excitation, and the light-sheet was generated using an achromatic cylindrical lens (ACY254, Thorlabs, Newton, NJ, USA) of 50-mm focal length. The sample was introduced into a quartz cuvette mounted on an *xyz* linear translational stage enabling automated acquisition of in-depth image stacks. The *x*- and *y*- axes correspond to the *xy* sample plane, while the *z*-axis corresponds to the sample depth. The main difference between SPIM systems was the optical collection path that imaged the cerebellum samples’ fluorescent planes.

For macro-SPIM, we used a high-magnification zoom lens system (Thorlabs MVL6X12Z; with an MVL20A extension tube) and a high-sensitive (CMOS) camera (Thorlabs DCC3240N). The laser light was filtered out from the fluorescence signal using a high-quality interferometric multiband optical filter (Em01-R488/568-25, Semrock, Rochester, NY, USA). The variable zoom lens magnification was from 1.4× to 9×. The FoV system was approximately 5.7 mm, covering around 4.5 × 3.4 mm^2^. The maximum optical resolution for this system is around 4 μm. Stacks composed of 3600 images of cerebellum fluorescent planes were taken, covering an approximate volume of 4.5 × 3.4 × 3.6 mm^3^ (width × height × depth). Each plane was pictured every 8 μm-depth, and the image size was 640 × 512 pixels^2^. Therefore, 5 GB files of raw data were stored for every stack using a Dell Precision T5810BTX computer (Intel^®^ Xeon^®^ Processor E5-1650 v3, 3.50 GHz), 16 GB 2133 MHz, 2 × 1 TB 7200 RPM 3.5” SATA Disks with Windows 64-bit operating system.

For micro-SPIM, the cerebellum fluorescent planes were imaged using an infinity-corrected objective lens (10×; NA: 0.25; WD: 13 mm, Olympus, Tokyo, Japan), a tube lens (Thorlabs TTL200) of 200-mm focal length, and a CMOS camera along with the high-quality interferometric multiband filter. Here, the obtained images possessed higher optical resolution (~2 μm). The different sample quadrants were stack-imaged (every 5 μm-depth) and stitched afterward to build a mosaic image of the region of interest. A total of 9996 planes of 640 × 512 pixels^2^ were acquired, covering an approximate volume of 3.5 × 3 × 0.7 mm^3^ (width × height × depth), generating 10 GB of raw data. The approximate capture time was 150 min.

#### 2.5.2. Image Processing and Reconstruction

Fluorescent image processing, focal plane concatenation, and 3D mosaic images were carried out using an Image-J Fiji software script. The complete mosaic reconstruction in 2D was done using Grid/Collection stitching [25] and was performed in two sections: column alignment and row alignment. The alignment per column was performed by pairing the top quadrants corresponding to each row, using the Pairwise stitching plugin of the Image J v.140 software, which uses a linear combination in the overlapping area, gently adjusting the intensity between the two images. Row alignment was performed with the Grid, a row-by-row plugin of Image J v.140 that uses the overlapping area by rows according to the designated splice points.

### 2.6. Golgi Cox Staining

Transgenic GFAP-eGFP P5–P7 male mice were anesthetized intraperitoneally with pentobarbital (Cheminova, 30 mg/kg) and perfused with 0.9% NaCl followed by a 4% PFA solution. After perfusion, brains were removed and kept in Golgi-Cox solution for 4 weeks at room temperature in the dark. 250 μm coronal sections from the cerebellum were obtained in a vibratome; then the sections were dehydrated with increasing amounts of ethanol and xylene. Finally, the tissue was placed on slides and covered with Permount mounting medium and dried for 3 days before observation under a light microscope [26]. Several images of different focal depths were obtained with an optical microscope (BX60, Olympus, Tokyo, Japan). The images were processed with the Helicon Focus software testing three focusing algorithms: weighted means (method A), depth map (method B) and pyramid (method C) according to the depth of field used. This allowed us to reconstruct high-resolution images. The processed images were binarized and the following parameters were evaluated: (1) diameter and area of the soma and (2) number and length of the processes. The reconstructed cells were evaluated using NeuronJ, an ImageJ plugin, which allows to trace and measure, the number and length of glial processes. For the analysis, several images of different focal depths (z-stack) were obtained, passing through individual stacks when it was necessary to distinguish between two processes that seem to overlap in the maximum projection. Therefore, cells in which the processes could not be differentiated by focal plane were not included in this analysis.

Then, the cells were drawn by hand, using a Camera Lucida application of the iOS operating system. For all data, the arithmetic mean, standard deviations, and mean standard error of the mean were calculated.

### 2.7. Calcium Imaging

#### 2.7.1. Slice Preparation

Transgenic GFAP-eGFP P5–P7 male mice were euthanized by decapitation after intraperitoneal anesthesia with sodium pentobarbital (Cheminova) at a dose of 30 mg/kg. After decapitation, the brain was rapidly removed and kept in calcium-free artificial cerebrospinal fluid solution (ACSF, pH 7.4) on ice and kept bubbling in 95% O_2_ and 5% CO_2_. The ACSF solution contained: NaCl [134 mM]; [2.5 mM] KCl; [2 mM] CaCl_2_; [1.3 mM] MgCl_2_; NaHCO_3_ [26 mM]; [1.25 mM] K_2_HPO_4_ and [10 mM] glucose [27].

Coronal sections of the cerebellum (250 μm in thickness) were obtained with a vibratome containing calcium-free ACSF solution on ice. Before incubation, slices were left at least 30 min in ACSF oxygenated solution at room temperature. Slices were incubated in the dark for 30 min at 37 °C, continuously oxygenated with 95% O_2_ and 5% CO_2_, loaded with the calcium indicator Calcium Orange (AM) (ThermoFisher Scientific, Waltham, MA, USA) dissolved in DMSO with 20% pluronic acid-127 (Molecular Probes, Eugene, OR, USA) for a final concentration of 10 µM [27,28,29]. The slices were placed in a perfusion chamber at room temperature with constant oxygenated ACSF solution at a flow rate of 4–5 mL/min. To prevent movement of the slice, a platinum wire grid was placed over it [27].

#### 2.7.2. Primary Culture of Astrocytes

CD1 P5–P7 male mice were euthanized by decapitation after intraperitoneal anesthesia with sodium pentobarbital (Cheminova) at a dose of 30 mg/kg. After decapitation, the brain was quickly removed. The cerebellum was kept in ACSF on ice (pH 7.4). The ACSF solution contained: [150 mM] NaCl; [5.4 mM] KCl; [2 mM] CaCl_2_; [1 mM] MgCl_2_; [5 mM] HEPES and [10 mM] glucose.

Isolated cells were obtained by mechanical dissociation with a fire-polished Pasteur pipette tip previously treated with silicone (sigmacote, Sigma). The cell suspension was placed in 1 mL of Dulbecco’s modified Eagle culture medium (DMEM) supplemented with 10% fetal bovine serum and 100 UI/mL penicillin-100 μg/mL streptomycin [30].

The cells were suspended in DMEM, and a 150 µL aliquot was taken from the middle part of the suspension and placed on a Petri dish containing coverslips (22 × 22 mm) previously treated with poly-l-lysine (Sigma Aldrich, Saint-Louis, MO, USA). DMEM (300 µL) was added to the Petri dish and incubated at 37 °C for 24 h. After 24 h, the medium was replaced by neurobasal medium (Invitrogen, Carlsbad, CA, USA) supplemented with 100 μL of G5, 25 μL of glutamine [200 mM] and 100 UI/mL penicillin-100 μg/mL streptomycin to favor differentiation, growth and maintenance of astrocytes [31,32].

Before calcium imaging, astrocytes at 5, 6 and 7 DIV were loaded with the calcium-sensitive fluorescent dye Fluo-4 AM (ThermoFisher Scientific, Waltham, MA, USA) dissolved in DMSO with 10% pluronic acid-127 (Molecular Probes, Eugene, OR, USA) for a final concentration of 5 µM in dark conditions [33]. Coverslips containing the astrocytes were placed in a batch chamber in constant perfusion with oxygenated ACSF solution at a flow rate of 4–5 mL/min at room temperature.

#### 2.7.3. Data Acquisition

[Ca^2+^]_i_ imaging was performed in a fluorescence microscope with a 20× water immersion objective (Olympus, 1.00 NA, 2.0 mm WD). Imaging was carried out at 488 and 549 nm excitation. Image sequences were collected using a PCO 4.2 sCMOS camera: 4 fps, images per video: 1680–2400 images, exposure time: 250 ms, recording time: 420–600 s and resolution of 1024 × 1024 pixels [34]. At the end of the experiment, the total number of cells loaded in the field was determined with a puff of ATP (100 mM). The total number of cells that responded to ATP (active and silent during the experiment), were considered as 100% [35].

#### 2.7.4. Data Analysis

The image sequences were processed using Fiji-ImageJ software and the analysis to examine the spatial and temporal changes in fluorescence (Ca^2+^ signal) was carried out using a set of MATLAB routines (Matlab 2018a, Asheboro, NC, USA). The ImageJ plugins Template Matching and Turbo Reg were used for assessing and removing motion artifacts of the image sequences. Subsequently, the background fluorescence was digitally subtracted from the raw data and ROIs were manually selected around the soma of the eGFP-positive and ATP-responsive cells. This selection was saved and superimposed on the image sequence of basal activity. A Matlab script was written to determine the Ca^2+^ signal for each ROI calculated by ΔF = (Fb − F0)/F0, where Fb was the normalized signal and F0 was the average of fluorescence during resting state from the image stack in a time window of 30 s, selected when there was no significant change in fluorescence (calcium transient event). Data were plotted and cells that showed spontaneous calcium transients were selected. Peaks in fluorescence were considered a transient calcium event if it exceeded 2.5 times the standard deviation of the baseline. For each experiment we determined: (1) number of cells with spontaneous activity per area, (2) number of events per cell in 7 or 10 min, (3) duration of each transient (in seconds). Also, for each slice preparation we determined the rise and fall kinetics of each transient. The rise time was defined as the time required to reach 10% to 90% of the maximum amplitude of each Ca^2+^ event, whereas the decay time was defined as the time required to fall 90% to 10% of the maximum amplitude of each Ca^2+^ event. For astrocytes in primary culture, we applied the interactive Fluorescence Single Neuron and Network Analysis Package (FluoroSNNAP) written in Matlab to compute the synchronization analysis and functional connectivity of the network of astrocytes [36].

### 2.8. Statistical Analysis

For all experiments, the data were represented as the mean ± standard error of the mean and statistical significance. To compare distributions of data sets, the Shapiro Wilk or Kolmogorov–Smirnov test was used. For determining the comparison between different metrics among the groups, we used parametric Student’s *t*-tests and non-parametric tests as appropriate. The * indicates significant difference (*p* < 0.05) and ** indicates significant difference (*p* < 0.001).

## 3. Results

### 3.1. Motor Coordination Impairment

To determine the effect of carmustine on behaviors associated with the cerebellum, we evaluated GFAP-eGFP male mice 30 days after they were born (P30) by the accelerated rotarod motor test, which is widely used to monitor altered cerebellar motor function [37,38,39]. Postnatal development of pups was monitored and slight differences in body weight were detected (Figure S1).

**Figure 1:**
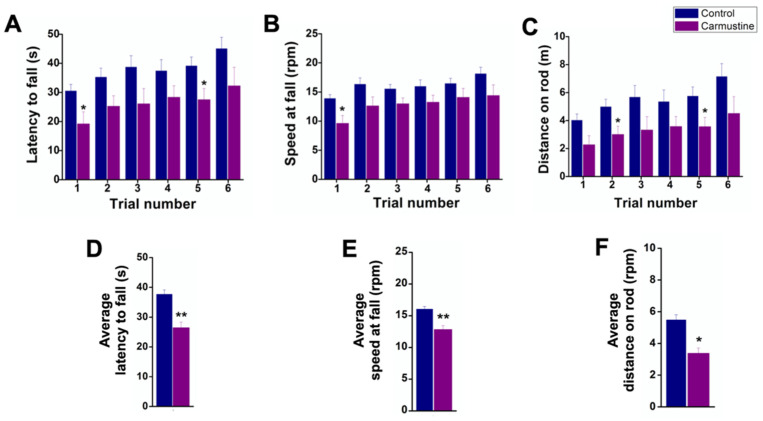
Deficiencies in motor coordination of carmustine-treated mice. Comparative analysis between control and carmustine-treated groups. (**A**). Mice treated with carmustine spent less time on an accelerating rotarod; this was significantly different in trials 1 and 5 (*p* < 0.05). (**B**). The speed at which the carmustine-treated group fell was slower in the six trials, but statistically significant only in trial 1 (*p* < 0.05). (**C**). The carmustine-treated group traveled a shorter distance throughout the test, but statistically significant in trials 2 and 5 (*p* < 0.05). (**D**). The average latency to fall is shown for each group (Ctrl 37.64 ± 1.49 s, Carmustine 26.45 ± 1.86 s) **. (**E**). The average speed at fall is shown for each group (Ctrl 16.03 ± 0.42 rpm, Carmustine 12.83 ± 0.59 rpm) **. (**F**) The average distance traveled is shown for each group (Ctrl 5.48 ± 0.31 m, Carmustine 3.26 ± 0.30 m) **. (Control *n* = 6, Carmustine *n* = 6). Data are represented as the mean ± the standard error of the mean. * indicates significant difference (*p* < 0.05) and ** indicates significant difference (*p* < 0.001) both compared to the control group.

Carmustine-treated mice fell faster in the first and fifth trials on the Rotarod (Figure 1A). However, fall latency time was not significantly different in the sixth trial, indicating that mice had deficiencies in motor coordination during the first and fifth trials of the test since they spent less time on the rod (Ctrl 37.64 ± 1.49 s, Carmustine 26.45 ± 1.86 s) **. In addition, the speed at fall in carmustine-treated mice was slower in the first trial (Figure 1B). The control group reached maximum acceleration, whereas the carmustine-treated group fell at lower speed (Ctrl 16.03 ± 0.42 rpm, Carmustine 12.83 ± 0.59 rpm) **. The distance traveled by carmustine-treated mice was shorter in the second and fifth trial. The total distances recorded revealed that the control group traveled a longer distance (Ctrl 5.48 ± 0.31 m, Carmustine 3.26 ± 0.30 m) **. We did not observe differences in latency falls among groups, although a slight but significant difference was observed between control and carmustine-treated groups only in the first and sixth trial (Figure S2).

Finally, data pooled from the six trials of the three Rotarod tests were analyzed (Control *n* = 6, Carmustine *n* = 6). A Student’s *t*-test revealed significant differences (*p* < 0.001) in the average latency to fall (Figure 1D), average speed at fall (Figure 1E) and average distance on the rod (Figure 1F). Taken together, these data confirm that motor behavior was affected by carmustine treatment.

### 3.2. Cerebellar Cortical Dysplasia

**Figure 2:**
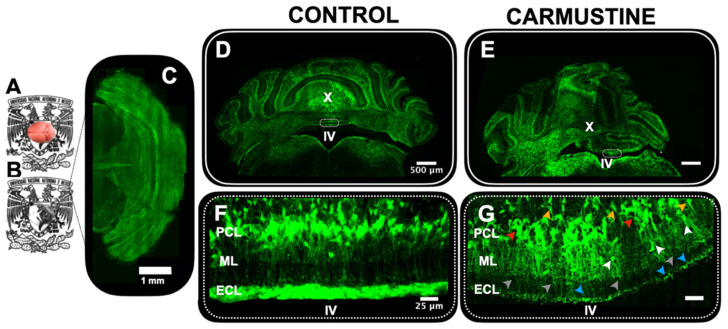
Morphological evidence of cortical dysplasia in the cerebellum. (**A**). Five-day-old male GFAP-eGFP transgenic mouse brain, before (**A**) and after clarification (**B**,**C**). Dorsal view of the brain in (**B**) reconstructed by light sheet microscopy. (**D**). Coronal section of the cerebellum of a male P5 GFAP-eGFP transgenic control mouse. (**E**). Coronal section of the cerebellum of a male P5 GFAP-GFP transgenic mouse treated with carmustine showing the presence of cortical dysplasias in the lobules in contact with the IV ventricle (**F**). Magnification of the square in (**D**); here Bergmann cells are situated in the Purkinje cell layer, projecting their processes through the molecular layer. (**G**). Magnification of the square in (**E**), where Bergmann cells are seen displaced towards the granular layer (orange arrowheads) and the molecular layer (white arrowhead) with disrupted processes (gray arrowhead); as well as the presence of cell clusters and heterotopias evidenced in the Purkinje cell layer (red arrowhead) and ependymal cell layer (blue arrowhead). (Control *n* = 5, Carmustine *n* = 5) Green: GFAP + glial cells. ML: molecular layer, PCL: Purkinje cell layer, GCL: granule cell layer, ECL: ependymal cell layer.

To investigate the extent of the effect of carmustine in the cerebellum, we analyzed the offspring of transgenic GFAP-eGFP male P5–P7 mice with light sheet fluorescence microscopy (Figure 1A–C). We collected data from five cerebella and determined that the most affected area was in contact with the fourth ventricle, consistent with previous findings [15]. We found that dysplasias experimentally induced by carmustine reproduced many morphological characteristics of cortical dysplasias in human pathological specimens (Figure 2D–G), including: (1) disruption of lamination, (2) heterotopias and (3) clusters of reduced morphological complexity [6,20]. Multiple heterotopic clusters of glial cells were observed, as well as the presence of BG cells and velate astrocytes displaced to the molecular and granular layers (Figure 2E). The disorganization of BG cells was evident: many somas were stratified in the granular layer and did not reach their normal position in the Purkinje cell layer; the processes were retracted and disorganized, projecting laterally and not positioned in the characteristic palisade oriented parallel to the longitudinal axis of the folia and perpendicular to the plane formed by the dendritic trees of the Purkinje neurons. The effect of carmustine was more evident on the roof of the fourth ventricle where lobule X is in contact with the cerebrospinal fluid as shown in the maximum projection images (Videos S2 and S4).

### 3.3. Carmustine Reduces the Morphological Complexity of Astrocytes and Bergman Cells

**Figure 3:**
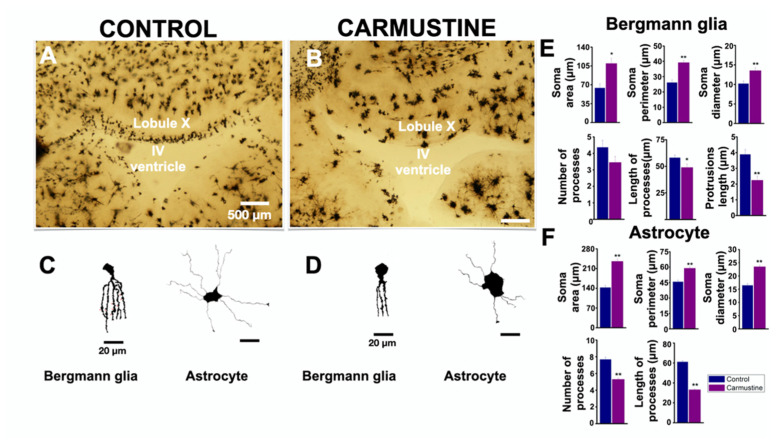
Carmustine treatment reduced morphological complexity of glial cells. (**A**). Coronal section at the level of lobule X of the cerebellum processed by Golgi-Cox staining. (**B**). A sample image of the same region showing a cortical dysplasia. Notice the malformation of the shape of the fourth ventricle. (**C**,**D**). Representative drawings with camera lucida of a Bergmann cell of lobule X and an astrocyte from cerebellar cortex, control and carmustine respectively. Complexity and extension of the astrocyte processes are observed in the control group, which contrasts with the carmustine-treated group, as fewer and diffuse processes are observed, as well as an increase in the size of the soma. (**E**,**F**). Comparative analysis between the control and the carmustine-treated groups (1 to 3 BG cells were analyzed from each experiment; Control *n* = 6, Carmustine *n* = 6 and 6 to 8 astrocyte cells were analyzed from each experiment; Control *n* = 6, Carmustine *n* = 6). Data are represented as the mean ± the standard error of the mean. * indicates significant difference (*p* < 0.05) and ** indicates significant difference (*p* < 0.001) both compared to the control group.

BG cells were evidently affected by the carmustine treatment as observed in Figure 2. This may suggest that other glial cells, such as astrocytes, may also be altered by the treatment. Thus, to determine if this damage is extended to astrocytes, we prepared histological sections for Golgi-Cox staining. This procedure confirmed the prevalence of cortical dysplasia at the roof of the fourth ventricle and revealed an increase in the size of the soma and an atrophy in the complexity of BG cells and astrocyte processes (Figure 3). The morphology of BG and astrocytes showed changes in their soma and processes, which were retracted and diffuse (Figure 3C,D), similar to that reported previously at postnatal age P5 [20], therefore, we measured different characteristics of these cells: BG showed significant differences for soma diameter (Ctrl 10.20 ± 0.68 μm, Carmustine 13.55 ± 0.72 μm) **, area (Ctrl 63.99 ± 7.95 μm, Carmustine 109.60 ± 9.33 μm) * and perimeter of the soma (Ctrl 26.14 ± 1.78 μm, Carmustine 39.20 ± 1.33 μm) **, as well as for process length (Ctrl 58.11 ± 2.56 μm, Carmustine 48.92 ± 3.09 μm) * and process protrusion length (Ctrl 3.88 ± 0.30 μm, Carmustine 2.24 ± 0.26 μm) **; while no significant difference was found in the number of processes (Ctrl 4.36 ± 0.43, Carmustine 3.45 ± 0.36) (1 to 3 BG cells were analyzed from each experiment, *n* = 6 for control mice and *n* = 6 for carmustine-treated mice). In astrocytes, the analysis showed that there is a significant difference for the soma diameter (Ctrl 16.42 ± 0.69 μm, Carmustine 23.48 ± 0.55 μm) **, area (Ctrl 142.65 ± 7.61 μm, Carmustine 253.79 ± 8.37 μm) ** and perimeter (Ctrl 45.76 ± 1.58 μm, Carmustine 58.95 ± 1.17 μm) **, as well as for the number (Ctrl 7.68 ± 0.26 μm, Carmustine 5.31 ± 0.14 μm) ** and length of the processes (Ctrl 61.04 ± 1.30 μm, Carmustine 33.29 ± 0.74 μm) ** (six to eight astrocyte cells were analyzed from each experiment, *n* = 6 for control mice and *n* = 6 for carmustine-treated mice). Data are represented as the mean ± the standard error of the mean. The * indicates significant difference (*p* < 0.05) and ** indicates significant difference (*p* < 0.001). Sample images of astrocytes and Bergman cells from both groups are shown in Figure S3.

### 3.4. Carmustine Induced a Higher Number of Bergmann Glia Engaged in Spontaneous [Ca^2+^]_i_ Oscillations

**Figure 4:**
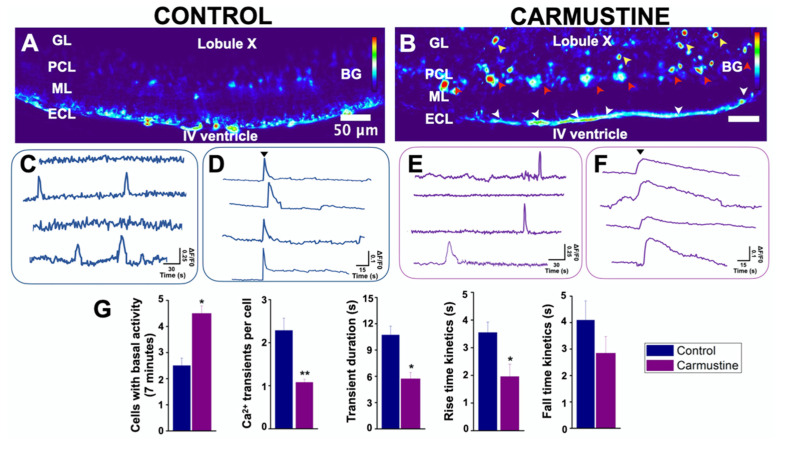
Basal activity of the Bergmann glia is increased in cortical dysplasia. (**A**,**B**). Heat maps of the maximum projection image of basal activity of the BG from a sample coronal section of the lobule X of the cerebellum of transgenic GFAP-eGFP male mice (P5). (**B**). The image reveals higher activity in the carmustine-treated cerebellum, mainly in BG cells (red arrowheads), astrocytes from the granular layer (yellow arrowheads), and ependymal cells (white arrowheads. (**C**,**E**). Representative basal (Ca^2+^) traces of control and carmustine-treated BG cells. More cells were engaged in calcium activity in the carmustine-treated animals. Notice the differences in the activity kinetics. (**D**,**F**). At the end of the recordings, application of ATP (black arrowhead) increased the (Ca^2+^) fluorescence (dF/F0) response in all BG. (**G**). Comparative analysis between the control and carmustine-treated groups. BG cells from carmustine-treated mice presented more cells with spontaneous Ca^2+^ activity, but with a reduced number of Ca^2+^ transient events per cell. BG also exhibited shorter duration and reduced rise-and-fall kinetics time in these events. An average of 83.5 ± 6.11 Bergmann cells responded to ATP in the recorded area of each experimental slice (Control *n* = 6), whereas 76.25 ± 2.39 Bergmann cells responded to ATP in carmustine-treated slices (Carmustine *n* = 5 pups, each from a different carmustine-treated mother). Data are represented as the mean ± the standard error of the mean. * indicates significant difference (*p* < 0.05) and ** indicates significant difference (*p* < 0.001) both compared to the control group. ML: molecular layer, PCL: Purkinje cell layer, GL: granule cell layer, ECL: ependymal cell layer.

Glial cell excitability is measured relative to intracellular calcium signaling. Since BG cells were morphologically affected by the carmustine treatment, it was predicted that changes in their communication could have occurred. BG cells generate spontaneous transients of calcium concentrations which are known to modulate the functional interactions with PC [40,41]. To test if the morphological changes of BG cells are related to functional characteristics, we determined the basal activity of the intracellular calcium dynamics of this group of cells. Changes in calcium activity were determined in 7-min image recordings of cerebellar slices loaded with Oregon orange, in which we observed little activity in BG cells (Figure 4, Control), in line with previous observations [34,39,40,41]. Most of the BG cells remained inactive during the recordings and only a few (2.5 ± 0.28 cells from 83.5 ± 6.11 in the control slice and 4.5 ± 0.57 cells from 76.25 ± 2.39 cells in the carmustine-treated slice) were active. At the end of the recordings, we applied adenosine triphosphate (ATP), which evoked (Ca^2+^) fluorescence (dF/F0) in all BG cells, thus establishing the viability of the slice and the ability of BG cells to evoke calcium activity. Figure 4 shows a sample heat map where red represents higher calcium activity as a change in the intensity of fluorescence, while blue represents the minimum value of change. The image reveals higher activity in the carmustine treated cerebellum, mainly in BG cells, astrocytes from the granular layer and ependymal cells (Figure 4B).

The comparative analysis between the control group and the carmustine-treated group for each of the evaluation criteria showed that more BG cells engage in spontaneous activity (Ctrl 2.5 ± 0.28, Carmustine 4.5 ± 0.57) *, a reduced number of intracellular Ca^2+^ transient events per cell (Ctrl 2.28 ± 1.06, Carmustine 1.07 ± 0.07) **, reduced duration of transient events (Ctrl 10.71 ± 5.99 s, Carmustine 5.71 ± 2.83 s) * and rise-and-fall kinetics of each transient (rise kinetics, Ctrl 3.54 ± 0.37, Carmustine 1.95 ± 0.45 s *; fall kinetics, Ctrl 4.09 ± 0.73, Carmustine 2.84 ± 0.63 s). Also, the Ca^2+^ response to ATP in Bergmann glia, showed significant differences in the comparative analysis between control and carmustine-treated group for transient duration: (Ctrl 3.8 ± 0.37417 s, Carmustine 25.8 ± 4.2 s) ** (Figure S4A). An average of 83.5 ± 6.11 Bergmann cells were recruited in activity in the recorded area of each experimental slice (*n* = 6 pups of control mice), whereas 76.25 ± 2.39 Bergmann cells were recruited in carmustine-treated slices (*n* = 5 pups, each from a different carmustine-treated mother). Data are plotted in Figure 4G represented as the mean ± standard error of the mean. The * indicates significant difference (*p* < 0.05) and ** indicates significant difference (*p* < 0.001) compared to the control group. These results show that morphological changes in BG cells severely affect their functional properties; nonetheless, they remain responsive to ATP, a classic transmitter in glial cells.

### 3.5. Carmustine Induced a Reduction in the Complexity of Astrocyte Morphology Which Shows More Spontaneous [Ca^2+^]_i_ Oscillations In Vitro

**Figure 5:**
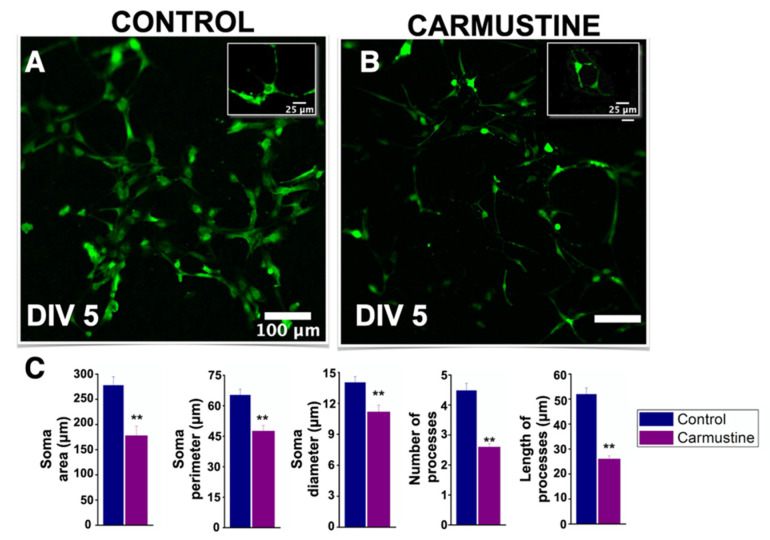
Decreased morphological complexity of carmustine treated astrocytes in vitro. (**A**,**B**). Astrocytes from control and carmustine-treated mice. The morphological complexity and extent of astrocyte processes are evident in this representative image at DIV5. (**B**). Astrocytes from carmustine treated mice showed a reduced number of processes that were retracted. (**C**). Comparative analysis between the control group and the carmustine-treated group for each parameter: soma area, soma perimeter, soma diameter, number of processes and length of the processes. (3 to 5 astrocyte cells were analyzed from each experiment; Control *n* = 4, Carmustine *n* = 4). Data are represented as the mean ± standard error of the mean. ** indicates significant difference (*p* < 0.001).

To assess the effect of carmustine on astrocytes we used primary cultures of cerebellar astrocytes isolated from the offspring of CD1 mice and evaluated the dynamics of [Ca^2+^]_i_ basal activity in vitro. First, similar to the histological sections, astrocytes in culture at DIV5 showed a reduction in their morphological complexity, soma diameter (Ctrl 14.02 ± 0.56 μm, Carmustine 11.41 ± 0.50 μm) *, soma area (Ctrl 277.73 ± 17.11 μm, Carmustine 189.46 ± 20.72 μm) *, and soma perimeter (Ctrl 65.26 ± 2.85 μm, Carmustine 50.56 ± 2.53 μm) * as well as in the number (Ctrl 3.71 ± 0.20 μm, Carmustine 2.80 ± 0.16 μm) * and length of the processes (Ctrl 50.79 ± 2.31 μm, Carmustine 26.69 ± 1.28 μm) ** 3 to 5 astrocyte cells were analyzed from each experiment; Control *n* = 4, Carmustine *n* = 4) (Figure 5).

**Figure 6:**
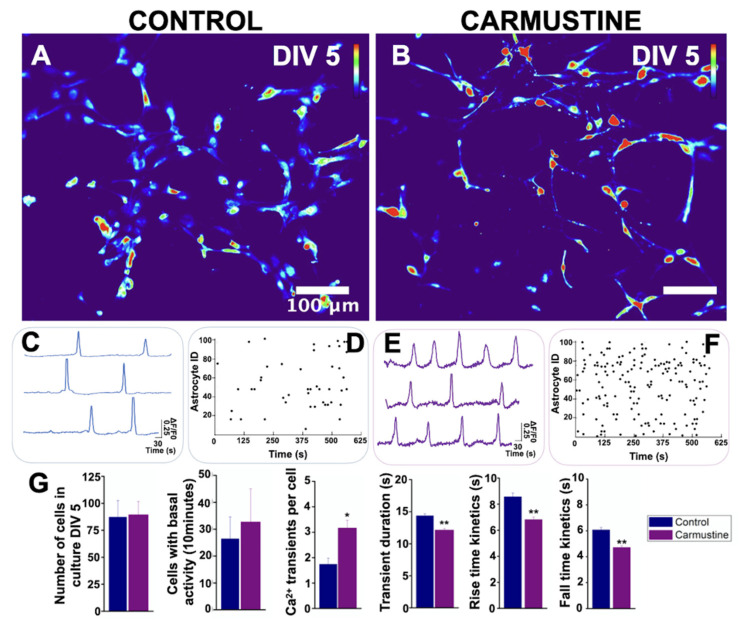
Basal activity of cerebellar astrocytes in culture. (**A**,**B**). Heat map of the maximum projection images of basal activity from primary cultures of cerebellar astrocytes at DIV5. Representative traces of basal activity of (**C**) control and (**E**) carmustine-treated mice. Contrast of the higher activity of astrocytes from carmustine treated mice. Raster plot of the basal activity of (**D**) control and (**F**) carmustine-treated astrocytes. The baseline activity of individual astrocytes is shown over time. The Y axis corresponds to the ID of each astrocyte and a dot indicates the time in which a calcium transient event occurred. (**G**). Comparative analysis between the control and the carmustine-treated group. Astrocytes from carmustine-treated mice culture presented more cells with spontaneous Ca^2+^ activity, with a higher number of Ca^2+^ transient events per cell. Astrocytes also showed shorter duration and reduced kinetics of rise and fall time of these events. An average of 87.25 ± 15.36 astrocytes responded to ATP at DIV5 culture, Control *n* = 4, 8 pups were used, 2 for each control culture; whereas 89.50 ± 12.29 astrocytes responded to ATP at DIV 5, Carmustine *n* = 4, 8 pups, 2 for each carmustine culture). Data are represented as the mean ± the standard error of the mean. * indicates significant difference (*p* < 0.05) and ** indicates significant difference (*p* < 0.001) both compared to the control group.

Next, we determined the intracellular [Ca^2+^]i dynamics. Unexpectedly, although the complexity of the processes of astrocytes was reduced by carmustine treatment, we observed that these cells exhibited increased spontaneous activity in 10-min recordings (Ctrl 26.5 ± 8.06, Carmustine 32.75 ± 12.25) and showed a higher number of intracellular Ca^2+^ transient events per cell (Ctrl 1.74 ± 0.23, Carmustine 3.17 ± 0.29 events) *, reduced duration of transient events (Ctrl 14.36 ± 0.30 s, Carmustine 12.15 ± 0.23 s) * and kinetics of rise and fall of each transient (rise kinetics Ctrl 8.56 ± 0.29, Carmustine 6.81 ± 0.19 s) **; (fall kinetics Ctrl 6.07 ± 0.19, Carmustine 4.70 ± 0.14 s) **. In control cultures, 87.25 ± 15.36 astrocytes were recruited at DIV5 culture, *n* = 4) (8 pups were used, 2 for each control culture) whereas 89.50 ± 12.29 astrocytes were recruited at DIV 5, *n* = 4 (8 pups, 2 for each carmustine culture). Data plotted in Figure 6G are represented as the mean ± standard error of the mean. * indicates significant difference (*p* < 0.05) and ** indicates significant difference (*p* < 0.001) compared to the control group. Figure S5 shows a comparative analysis of cell morphology and calcium kinetics at 5-7 DIV. Together, these data suggest that astrocyte calcium activity is affected by carmustine treatment and that the effects remain even after the cells are cultured in vitro.

### 3.6. Astrocyte [Ca^2+^]_i_ Oscillations In Vitro Show a Disrupted Global Synchronization Network

**Figure 7:**
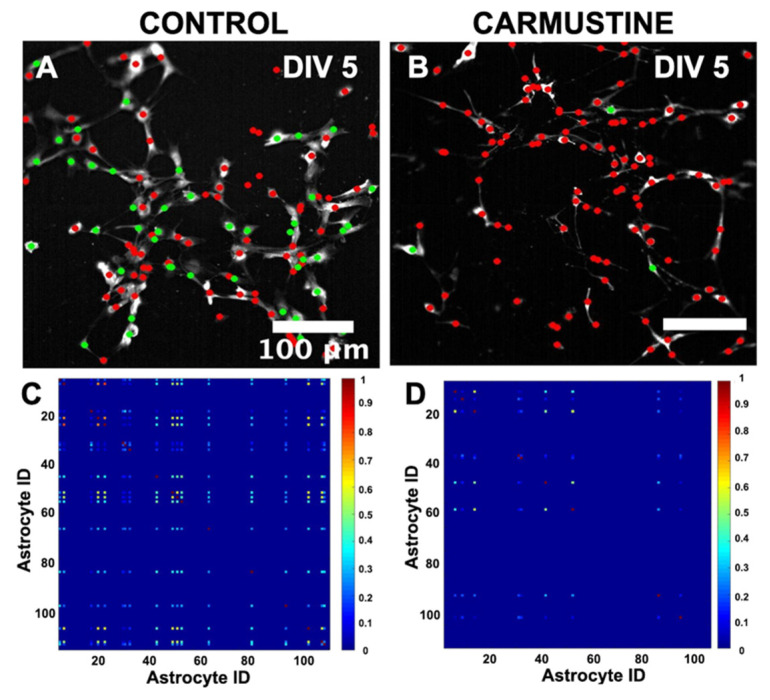
Global network synchronization in cerebellar astrocytes in culture. (**A**,**B**). Representative images of a pairwise synchronization matrix that show the coactive astrocytes exhibiting transient calcium events between all active astrocyte pairs. (**C**,**D**). Representative images of core ensemble, defined as a group of coactive astrocytes. Each astrocyte (colored circle) is a node. The nodes that are conserved in all significantly correlated ensembles are in green and the rest are in red. The core ensemble preserves the spatial distribution of the astrocytes in the culture. The calibration bar corresponds to the events in which astrocytes activated together. (87.25 ± 15.36 astrocytes were recruited at DIV5 culture, Control *n* = 4, 8 pups were used, 2 for each control culture, whereas 89.50 ± 12.29 astrocytes were recruited at DIV 5, Carmustine *n* = 4, 8 pups, 2 for each carmustine culture).

Astrocytes form a complex phenomenon of active networks of global synchronization in vitro [33,36]. To understand how the dynamics of [Ca^2+^]_i_ oscillations changed after carmustine treatment in the astrocytic network formed in vitro, we studied the interactions between the number of simultaneous calcium transient events over time in the same field of view, using the Fluorescence Single Neuron and Network Analysis Package (FluoroSNNAP) [36]. The basal activity patterns in carmustine-treated astrocytes at DIV5 showed a lower level of synchronization matrix (Ctrl 0.13 ± 0.02, Carmustine 0.05 ± 0.01) regardless of the increase in the frequency of calcium oscillations, which evidently are not in synchrony, since they act independently of neighboring cells, thus showing a sparse synchronization matrix (Figure 7D). This is in line with previous reports that indicated that global synchronization is disrupted in astrocytes with reduced complex morphology [33,41,42].

## 4. Discussion

By employing an experimental model of cortical dysplasia induced by carmustine, we have assessed the effects on the neuroglia of the cerebellum. During embryonic development, between E13 and E14, BG precursors are derived from radial glial cells in the ventricular zone [43]. We chose E13 to administer carmustine since this is the peak of gliogenesis which coincides with the increase of BG cells [5]. Previous studies showed that the mechanisms that give rise to cerebellar foliation and laminar organization depend on Zeb2 regulation of BG during cerebellum development [44]. Thus, changes in the differentiation of BG would disrupt cell migration, producing an abnormal stratification of the cell layers and altered spatial distribution causing malformations in the cerebellar cortex. As shown in our findings, cortical cerebellar alterations were observed in the whole cerebellum but most frequently in the lobules that are in contact with the fourth ventricle.

Analysis of the behavioral tests of carmustine-treated animals revealed impairment in coordination and locomotor activity, providing evidence for the impact of this treatment on cerebellar motor function but not on motor learning or the ability to learn the motor tasks (Figure 1). Defects and alterations in cerebellar cortical formation are two main causes of long-term cognitive and motor coordination deficits; preterm birth induced by c-sections in pigs reduced the development of BG cells and granule cell precursors [45]. In addition, preterm infants who died shortly after birth also showed decreased numbers of BG cells [46]. Thus, the impaired coordination and locomotor activity of carmustine-treated mice correlate with the changes in BG cell morphology and function in these animals.

Glial cells are electrically non-excitable, but display spontaneous transients of [Ca^2+^]_i_ in response to activation of specific membrane receptors [47]. The basal activity of cells is a complex phenomenon that depends on changes in the ionic currents, homeostasis of the extracellular environment and cell maturation [33]. In this study, we showed an increased basal activity of BG and astrocytes in carmustine-treated mice. We first evaluated the [Ca^2+^]_i_ responses from BG. Basal activity in BG was relatively low, and in many cells we did not detect responses at all during the recording time. These findings are in line with previous reports [40,41] that indicated that BG cell [Ca^2+^]_i_ oscillations are indeed infrequent regardless of the preparation. In contrast, more BG cells from carmustine-treated animals showed [Ca^2+^]_i_ signaling, furthermore, the duration of each event was reduced and the kinetics altered. This result was unexpected since the BG cells were morphologically affected, as evidenced in the Golgi-Cox preparation (Figure 3), showing an increase in the size of the soma and retraction of its processes. As mentioned before, calcium signaling depends on the activation of specific membrane receptors [47]. BG cells express glutamate AMPA receptors (a-amino-3-hydroxy-5-methyl-4-isoxazolepropionic acid), composed of the GluA1 and GluA4 subunits, and the inactivation of AMPA receptors in BG caused structural changes and retraction of processes, affecting the evoked PC currents accompanied by alterations in motor coordination [47,48]. This suggests that the retraction of glial processes affected the function of AMPA receptors, which are Ca^2+^ permeable, resulting in inefficient glutamate clearance, which in turn altered the synapses with Purkinje neurons [47,48].

In patients with intractable epilepsy associated with focal cortical dysplasia astrocytes show functional plasticity by increasing the expression of purinergic receptors (P2Y1, P2Y2, P2Y4), metabotropic glutamate receptors (mGluR1 and mGluR5) and potassium channels (Kv4.2 and Kir4.1) [11]. P2Y1 receptors are also expressed in BG cells, where they are the main target of ADP and ATP [49]. As such, responses to ATP are essential for the mobilization of Ca^2+^ from internal reserves and would be affected in our model due to the morphological changes induced by carmustine. In other instances, overexpression of P2Y1 receptors in astrocytes affect the activity of Ca^2+^ waves, such as in the cases of epilepsy, stroke and Alzheimer’s disease, which highlights the relevance of purinergic signaling to preserving the correct function of synaptic transmission [50,51,52,53,54,55].

We also evaluated the [Ca^2+^]_i_ responses from astrocytes in vitro. Astrocytes from carmustine-treated mice showed increased frequency of [Ca^2+^]_i_ oscillations and diminished global synchronization index at DIV5. A previous study reported that the absence of the ataxia telangiectasia mutated (ATM) protein in cerebellar astrocytes in culture reshapes their morphology, producing a reduction in the length and number of processes, which in turn negatively regulates the mammalian target of rapamycin (mTOR) pathway [33]. In addition, it has also been reported that manipulation of mTOR signaling disrupts the glial scaffold in primary cortical and organoid cultures [56]. These structural changes are associated with abnormal developmental maturation [33,35,36] which also alters calcium signaling and affects the cerebellar networks. These findings are consistent with previous reports that indicate that in neurological diseases astrocytes present aberrant calcium signal oscillations due to abnormal calcium homeostasis [36].

Astrocyte networks display synchronized recurrent activity, which is an important characteristic of dynamic systems, as it allows joining groups of functional assemblies to communicate more easily [33,36]. Although the astrocytes from mice treated with carmustine presented a higher frequency of calcium oscillations on DIV5, they also presented a lower occurrence of global network synchronizations. This may be due to the reduction of cell process length, which does not allow the formation of optimal functional connections with neighboring cells, thus disrupting the astrocyte functional network.

In the global analysis of the intracellular [Ca^+2^]_i_ dynamics in primary culture of cerebellar astrocytes at DIV6 and DIV7, the astrocytes of the group treated with carmustine remained in the same initial cell confluence (DIV5) and showed the same number of cells with basal activity. In contrast, the control astrocytes at DIV6 and DIV7 showed an increase in the frequency of spontaneous activity, followed by a plateau of coordinated activity and a higher index of global synchronization. This evolution in the network activity is consistent with previous studies that show that cells in culture undergo a process of morphological and physiological growth and maturation [36]. This suggests that carmustine treatment affected the development and maturation of the astrocytes, reducing their morphological complexity and thus impacting the calcium activity and level of global synchronization of the cells.

## 5. Conclusions

In the present study we show that administration of carmustine to pregnant mice induces cortical dysplasia in the offspring, altering the morphology and calcium signaling in Bergmann’s glia and astrocytes, which showed a reduced morphological complexity of the processes and cell soma. The morphological changes are associated with a higher frequency of spontaneous Ca^2+^ transients. Furthermore, we found a dramatic dysfunction of the astrocytic functional network in vitro that showed a lower index of global cell synchronization; this may be associated to a multiscale accumulation of structural and functional defects in the cerebellar circuits. Behavioral alterations linked to cerebellar function were affected, including motor coordination and increased fall latency in the rotarod. However, further studies are necessary to determine the molecular components altered in glial cells that lead to changes in neuronal function related to the behavioral changes.

## Data Availability

Data available upon request.

## Supplementary Materials

The following are available online at https://www.mdpi.com/article/10.3390/cells10071581/s1. Video S1. Control cerebellum reconstruction with macroSPIM. Maximum projection of GFAP-eGFP transgenic mouse cerebellum imaged with macroSPIM. Video S2. Carmustine cerebellum reconstruction with macroSPIM. Maximum projection of GFAP-eGFP transgenic mouse carmustine-cerebellum imaged with macroSPIM, showing the presence of cortical dysplasia mainly the lobule X, where the size of the ventricle is expanded and atrophied. Video S3. Fourth ventricle reconstruction with microSPIM. Coronal view of the roof of fourth ventricle show the normal pattern organization of Bergmann cells. Video S4. Carmustine fourth ventricle reconstruction with microSPIM. Coronal view of the roof of fourth ventricle show the spatial alteration of Bergmann cells. Figure S1. Early postnatal development of carmustine-treated mice at postnatal day 5 was monitored. The pups grew up without any apparent problem except for a lower body weight in comparison with the control animals (Ctrl 3.578 ± 0.043 g, Carmustine 3.142 ± 0.037 g) **. Also, after perfusion, the brain was extracted and their weight was examined during the postnatal ages P5, P10 and P21 for both groups. The brain weight of the carmustine-treated mice was lower during postnatal development for the different ages: P5 (Ctrl 0.234 ± 0.0074 g, Carmustine 0.183 ± 0.0065 g) *, P10 (Ctrl 0.378 ± 0.017 g, Carmustine 0.324 ± 0.005 g) * and P21 (Ctrl 0.416 ± 0.008 g, Carmustine 0.363 ± 0.004 g) * (Figure S1A). The length brain was measured, from the anterior end of the olfactory bulbs to the posterior end of the cerebellum. As in a previous analysis, the measurements were performed on brains from both groups during the same postnatal ages: carmustine-treated mice were smaller at P5 (Ctrl 9.261 ± 0.113 mm, Carmustine 8.147 ± 0.117 mm) **, P10 (Ctrl 10.92 ± 0.155 mm, Carmustine 9.44 ± 0.191 mm) ** and P21 (Ctrl 11.58 ± 0.211 mm, Carmustine 10.81 ± 0.204 mm) * (Figure S1B,C). Data are represented as the mean ± the standard error of the mean. * indicates significant difference (*p* < 0.05) and ** indicates significant difference (*p* < 0.001) both compared to the control group. Figure S2. An intragroup comparison in fall showed no significant differences between trials 1 to 6 for control (ANOVA F [5,30] = 1.95, *p* = 0.11519) (Figure S2A) and carmustine-treated mice (ANOVA F [5,30] = 0.86, *p* = 0.51536) (Figure S2B). Although we noticed a tendency in the carmustine-treated group to spend more time on the rod with each trial, there was not significant difference. However, significant differences were observed between control and carmustine-treated groups only in the first and sixth trial (Two-way ANOVA F [11,60] = 3.19, *p* = 0.01506) (Figure S2C). One-way analysis of variance followed by Tukey’s multiple comparison test and Two-way analysis of variance was performed. Figure S3. Sample images from Golgi-Cox impregnated cells from carmustine-treated and control cerebella. Figure S4. Ca^2+^ responses to ATP in Bergmann glia, showed significant differences lasted longer in the carmustine-treated mice: Ctrl 3.8 ± 0.37417 s, Carmustine 25.8 ± 4.2 s). Figure S5. Summary of cerebellar astrocytes in culture DIV5, DIV6 and DIV7. (A,B), (D,E) and (G,H). Primary culture of cerebellum astrocytes from a control and carmustine-treated male CD1 P2 mouse respectively in DIV5, DIV6 and DIV7 loaded with the calcium indicator Fluo4 AM. (C,F and I). Comparative analysis between the control group and the group treated with carmustine for each of the evaluation criteria. Data are represented as the mean ± standard error of the mean. The * indicates significant difference (*p* < 0.05) and ** indicates significant difference (*p* < 0.001) both compared to the control group.
